# TIM-4 is differentially expressed in the distinct subsets of dendritic cells in skin and skin-draining lymph nodes and controls skin Langerhans cell homeostasis

**DOI:** 10.18632/oncotarget.9546

**Published:** 2016-05-21

**Authors:** Xilin Zhang, Queping Liu, Jie Wang, Guihua Li, Matthew Weiland, Fu-Shin Yu, Qing-Sheng Mi, Jun Gu, Li Zhou

**Affiliations:** ^1^ Department of Dermatology, Second Military Medical University Changhai Hospital, Shanghai, China; ^2^ Henry Ford Immunology Program, Henry Ford Health System, Detroit, MI, United States of America; ^3^ Department of Dermatology, Henry Ford Health System, Detroit, MI, United States of America; ^4^ Department of Anatomy and Cell Biology, Wayne State University School of Medicine, Detroit, MI, United States of America; ^5^ Department of Internal Medicine, Henry Ford Health System, Detroit, MI, United States of America

**Keywords:** TIM-4, Langerhans cells, dendritic cells, skin, skin-draining lymph node, Immunology and Microbiology Section, Immune response, Immunity

## Abstract

T cell immunoglobulin and mucin-4 (TIM-4), mainly expressed on dendritic cells (DC) and macrophages, plays an essential role in regulating immune responses. Langerhans cells (LC), which are the sole DC subpopulation residing at the epidermis, are potent mediators of immune surveillance and tolerance. However, the significance of TIM-4 on epidermal LCs, along with other cutaneous DCs, remains totally unexplored. For the first time, we discovered that epidermal LCs expressed TIM-4 and displayed an increased level of TIM-4 expression upon migration. We also found that dermal CD207^+^ DCs and lymph node (LN) resident CD207^−^CD4^+^ DCs highly expressed TIM-4, while dermal CD207^−^ DCs and LN CD207^−^CD4^−^ DCs had limited TIM-4 expressions. Using TIM-4-deficient mice, we further demonstrated that loss of TIM-4 significantly upregulated the frequencies of epidermal LCs and LN resident CD207^−^CD4^+^ DCs. In spite of this, the epidermal LCs of TIM-4-deficient mice displayed normal phagocytic and migratory abilities, comparable maturation status upon the stimulation as well as normal repopulation under the inflamed state. Moreover, lack of TIM-4 did not affect dinitrofluorobenzene-induced contact hypersensitivity response. In conclusion, our results indicated that TIM-4 was differentially expressed in the distinct subsets of DCs in skin and skin-draining LNs, and specifically regulated epidermal LC and LN CD207^−^CD4^+^ DC homeostasis.

## INTRODUCTION

The T-cell immunoglobulin domain and mucin domain (TIM) family of genes, which contained eight members (encoding TIM-1 to TIM-4 and putative TIM-5 to TIM-8) in mice and three members (Tim-1, Tim-3 and Tim-4) in human, was first positionally cloned in the T cell and airway phenotype regulator (Tapr) locus as a novel group of allergy and asthma susceptibility genes [1, 2]. The evolutionarily-conserved TIM members share a similar structure of cell-surface Type 1 membrane protein, which is comprised of an N-terminal Cysteine-rich immunoglobulin variable-like domain, a mucin-like glycosylated domain, a transmembrane domain and an intracellular tail. Previous studies emphasized a substantial role of the TIM family during various immune responses, including viral infection, allergy, autoimmunity, transplant tolerance and neoplasm immunity [3–5]. The underpinning mechanisms are mainly postulated as their regulation of T cell polarization. The *in vivo* functions of TIM-1 were manifold: high-affinity TIM-1-specific antibody enhanced T helper cell 1 (Th1) and Th17 responses, but hampered regulatory T cell (Treg) differentiation; low-affinity TIM-1 engagement promoted Th2 polarization with compromised T cell proliferation [6]. And, TIM-2 and TIM-3 preferentially enhanced Th2 differentiation and inhibited Th1 differentiation, respectively [7, 8].

TIM-4, also termed as SMUCKLER (spleen, mucin-containing, knockout of lymphotoxin), was originally discovered by gene expression profiling in 2004 [9]. Being the only TIM member absent in T cells, TIM-4 was predominantly expressed in “professional” antigen-presenting cells (APC), including macrophages and conventional dendritic cells (DC) [10, 11]. Unlike the other TIM members, which contain an intracellular tyrosine phosphorylation motif, TIM-4 does not contain such motifs on the intracellular tail and cannot be phosphorylated upon T cell activation and subsequently transduce the signals [12, 13]. Nonetheless, TIM-4 displays pleiotropic and yet paradoxical immunoregulatory functions. As a costimulatory molecule on APCs, TIM-4 induced pre-activated T cell expansion by binding to TIM-1, while inhibited naïve T cell proliferation through an unidentified ligand other than TIM-1 [11, 14]. Additionally, TIM-4 expressed on oral mucosal DCs enhanced polarization of CD4^+^ T cells to Th2 phenotype, whereas splenic DC-expressed TIM-4 prevented induced Treg (iTreg) generation [15, 16]. However, as a phosphatidylserine (PS) receptor, TIM-4 on APCs mediated immune tolerance by the phagocytosis of antigen-specific T cells [10, 17–20]. Moreover, the aberrant persistence of apoptotic bodies in TIM-4^−/−^ C57BL/6 mice led to hyperactive T and B cells along with autoimmune manifestations [21]. Accordingly, TIM-4 dysregulation has been implicated in several autoimmune diseases, including systemic lupus erythematosus, rheumatoid arthritis and experimental autoimmune encephalomyelitis [22].

A versatile and heterogeneous group of DCs, residing in the skin and its draining lymph nodes (LN), are essential mediators of immunity and tolerance [23]. Epidermal Langerhans cells (LC), which characteristically express C-type lectin langerin (CD207), represent the prototype of cutaneous DCs [24]. LCs capture and present external or internal antigens to naïve T cells within the skin-draining LNs, where they secrete cytokines and provide co-stimulatory signals to induce either immunogenic or tolerogenic immune response [25]. Previous studies have demonstrated a pivotal role of LCs during T cell polarization that they are capable of selectively inducing Th1, Th2 and Th17 priming as well as Treg expansion under different stimuli [26–29]. Other DC subsets in the skin and its draining LNs, which differ in phenotype and function, are also potent immune modulators [30–32]. Concomitantly, these DCs contribute in the pathogenesis of various skin diseases, including infection, allergy, autoimmunity and neoplasm [33–38]. Therefore, identifying the key regulators of skin-related DCs would benefit the development of new therapeutic measures. Recently, Yeung *et al* reported that *in vivo* blockade of TIM-4 promoted skin allograft survival by conversion of naïve CD4^+^ T cells to allospecific iTregs [16]. Given the indispensable role of skin-related DCs in mediating regional immunity, we hypothesized that TIM-4 might regulate their homeostasis and function.

In this study, we sought to examine the expression pattern and immune function of TIM-4 in the DCs locating at the skin and skin-draining LNs, with a special focus on epidermal LCs. For the first time, we demonstrated that TIM-4 was differentially expressed in the distinct subsets of DCs within the skin and skin-draining LNs. Particularly, TIM-4 was expressed on epidermal LCs at a low level and its expression was upregulated upon migration, whereas dermal CD207^+^ DCs (CD207^+^ DDC), LN CD207^−^CD4^+^CD8^−^ and CD207^−^CD4^+^CD8^+^ DCs highly expressed TIM-4. TIM-4 deficiency led to an increased ratio of epidermal LCs with no impairment of their maturation, phagocytosis, migration and repopulation abilities, but the dinitrofluorobenzene (DNFB)-induced contact hypersensitivity (CHS) response remained intact. In the skin-draining LNs, lack of TIM-4 increased the frequencies of CD207^−^CD4^+^CD8^−^ and CD207^−^CD4^+^CD8^+^ DCs while downregulated the percentage of CD207^−^CD4^−^CD8^−^ DCs.

## RESULTS

### TIM-4 is differentially expressed by the distinct subsets of cutaneous DCs

**Figure 1:**
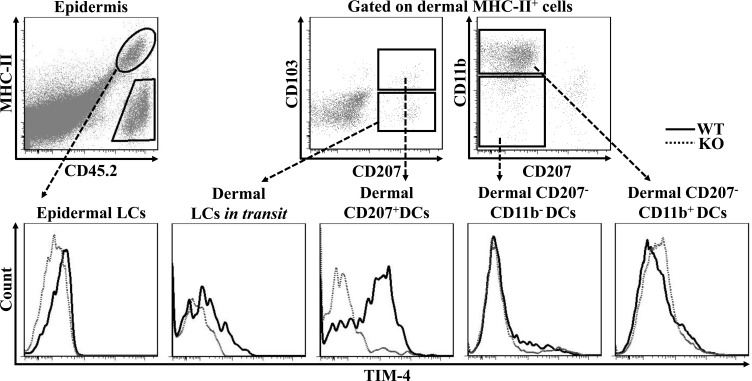
TIM-4 is differentially expressed by the distinct subsets of cutaneous DCs Epidermal and dermal cells were freshly-isolated from the skin TIM-4 WT and KO mice. Representative FACS dot plots for epidermal LC (MHC-II^+^CD45.2^+^), dermal LCs *in transit* (MHC-II^+^CD207^+^CD103^−^), dermal CD207^+^ DCs (MHC-II^+^CD207^+^CD103^+^), dermal CD207^−^CD11b^−^ DCs (MHC-II^+^CD207^−^ CD11b^−^) and dermal CD207^−^CD11b^+^ DCs (MHC-II^+^CD207^−^CD11b^+^) (upper panel). The MFI of TIM-4 expression in each cutaneous DC subset (lower panel). All data represent one of at least four independent experiments, with 2-5 mice per experiment.

While LCs represent the only DC subset within the epidermis, several DC subpopulations coexist in the steady-state dermis, including CD207^+^ DDCs, CD207^−^CD11b^−^ DDCs, CD207^−^CD11b^+^ DDCs and LCs *in transit*, which are the migrated LCs (mLC) enroute from the epidermis to nearby draining LNs [39, 40]. To explore a possible regulatory role of TIM-4 in cutaneous DCs, we first assessed its expression in the different DC subpopulations. As shown in Figure 1, epidermal LCs barely expressed TIM-4; however, dermal LCs *in transit* had a clear increase of TIM-4 expression. Interestingly, among other dermal DC subsets, CD207^+^ DDCs highly expressed TIM-4 whereas CD207^−^CD11b^−^ and CD207^−^CD11b^+^ DDCs did not express TIM-4 (Figure 1). Thus, TIM-4 exhibits preferential expression in CD207-positive cutaneous DCs.

### Disparate expressions of TIM-4 in the different subsets of LN DCs

**Figure 2:**
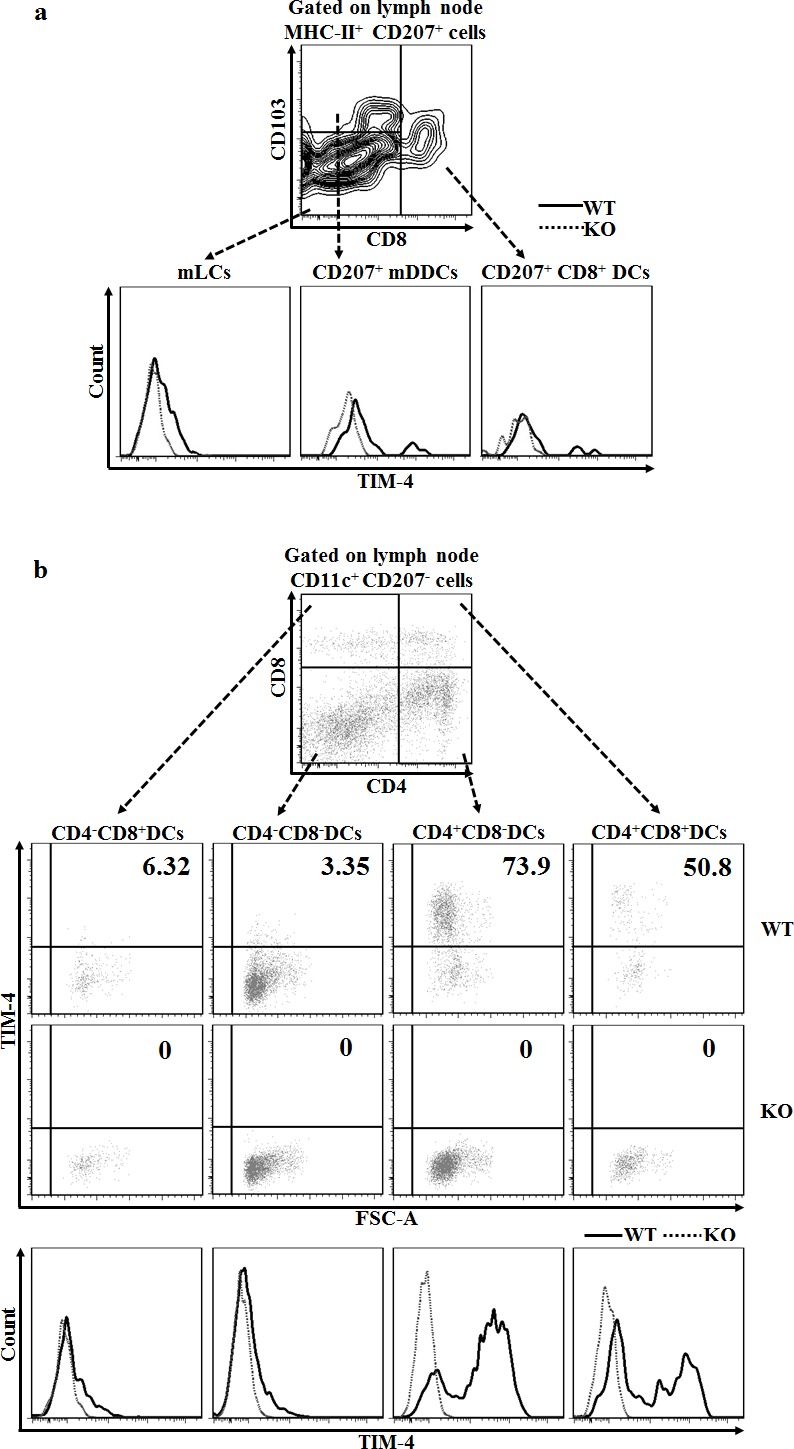
Disparate expressions of TIM-4 in the different subsets of skin-draining LN DCs LN cells were harvested from the skin-draining LNs of TIM-4 WT and KO mice. **a.** Representative FACS dot plots for LN mLCs (MHC-II^+^CD207^+^CD8^−^CD103^−^), CD207^+^ mDDCs (MHC-II^+^CD207^+^CD8^−^CD103^+^) and CD207^+^CD8^+^ DCs (MHC-II^+^CD207^+^CD8^+^) (upper panel). The MFI of each LN DC subset (lower panel). All data represent one of three independent experiments, with 3-5 mice per experiment. **b.** Representative FACS dot plots for the LN CD4^−^CD8^+^ DCs, CD4^−^CD8^−^ DCs, CD4^+^CD8^−^ DCs and CD4^+^CD8^+^ DCs gated on CD11c^+^CD207^−^ LN cells (upper panel). Representative FACS analysis of TIM-4 expression (middle panel) and MFI (lower panel) of each DC subset. All data represent one of two independent experiments, with 3-4 mice per experiment.

The skin-draining LNs contain two major populations of DCs: One subset is migratory DCs, which include epidermal LCs and CD207^+^ DDCs; the other subpopulation is tissue-resident DCs, which encompass CD207^+^CD8^+^ DCs and a group of conventional CD207^−^ DCs subcategorized based on the expression of CD4 and CD8 [41–43]. As depicted in Figure 2a, LN mLCs maintained a similar level of TIM-4 expression compared to their dermal counterparts LCs *in transit*, whereas migrated CD207^+^ DDCs (CD207^+^ mDDC) slightly decreased their TIM-4 expression upon migration. Additionally, tissue-resident CD207^+^CD8^+^ DCs displayed a moderate level of TIM-4 expression (Figure 2a). Remarkably, the CD207^−^CD4^+^CD8^−^ and CD207^−^CD4^+^CD8^+^ DCs highly expressed TIM-4 while the remaining CD207^−^CD4^−^CD8^−^ and CD207^−^CD4^−^CD8^+^ DCs had a much lower TIM-4 expression (Figure 2b). In brief, TIM-4 is wildly expressed in all the CD207-positive DC subsets of skin and skin-draining LNs, although LCs and CD207^+^ DDCs upregulate and downregulate the expression of TIM-4 upon migration, respectively; as for LN conventional CD207^−^ DCs, TIM-4 is preferentially expressed in the CD4-positive subpopulations.

### Loss of TIM-4 increases the ratio of epidermal LCs

**Figure 3:**
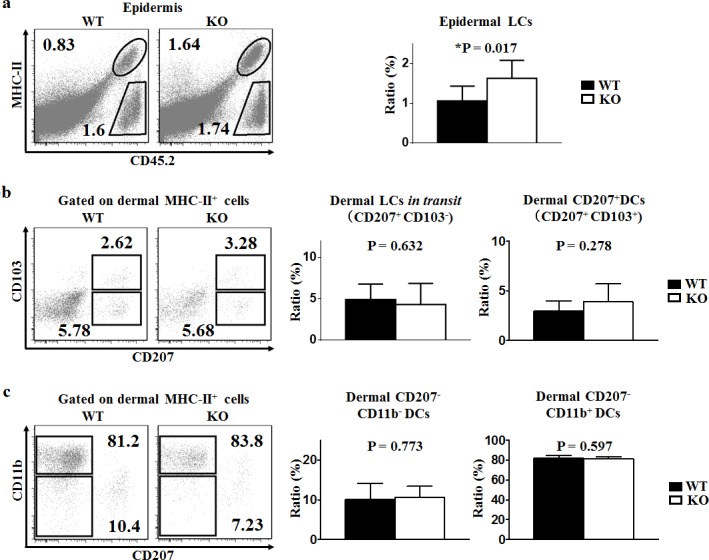
TIM-4 deficiency increases the ratio of epidermal LCs Epidermal and dermal cells were freshly-isolated from the skin of TIM-4 WT and KO mice. **a**.-**c**. Representative FACS analysis of epidermal LCs (a), dermal LCs *in transit* and dermal CD207^+^ DCs (b), dermal CD207^−^CD11b^−^ DCs and dermal CD207^−^CD11b^+^ DCs (c) from the trunk skin of TIM-4 WT and KO mice (left panel). The ratios of different skin DC populations in TIM-4 WT and KO mice (right panel). Data represent the results of at least four independent experiments, with 2-5 mice per experiment.

Using C57BL/6 wild-type (TIM-4 WT) and TIM-4^−/−^ C57BL/6 knock-out (TIM-4 KO) mice, we found that TIM-4 deficiency significantly increased the ratio of epidermal LCs (1.10 ± 0.39% in WT mice vs. 1.63 ± 0.48% in KO mice, P = 0.017) (Figure 3a) but did not affect the frequency of dermal LCs *in transit* (Figure 3b). The ratios of CD207^+^ DDCs, CD207^−^CD11b^−^ and CD207^−^CD11b^+^ DDCs in TIM-4 KO mice were all within normal ranges, and were comparable to that of TIM-4 WT mice (Figure 3b, 3c) [41].

### Loss of TIM-4 upregulates the ratios of LN CD207^−^CD4^+^CD8^−^ and CD207^−^CD4^+^CD8^+^ DCs

**Figure 4:**
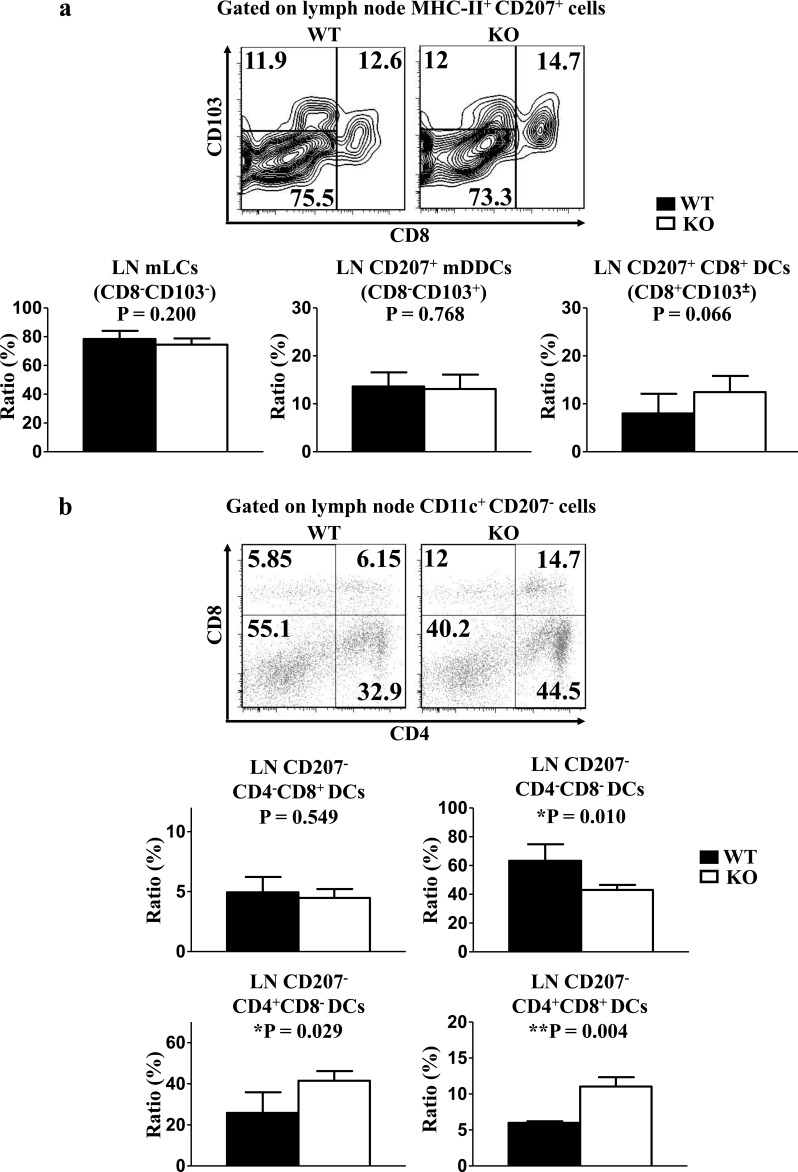
Lack of TIM-4 upregulates the frequencies of LN CD207-CD4^+^ DCs LN cells were harvested from the skin-draining LNs of TIM-4 WT and KO mice. **a.** Representative FACS analysis of LN mLCs, CD207^+^CD8^+^ DCs, CD207^+^ mDDCs from skin-draining LNs of TIM-4 WT and KO mice (upper panel). The ratios of different LN DC populations (lower panel). Data represent the results of three independent experiments, with 3-5 mice per experiment. **b.** Representative FACS analysis of LN CD207^−^CD4^+^CD8^−^ DCs, CD207^−^CD4^+^CD8^+^ DCs, CD207^−^CD4^−^CD8^+^ DCs and CD207^−^CD4^−^CD8^−^ DCs from skin-draining LNs of TIM-4 WT and KO mice (upper panel). The ratios of different LN DC populations (lower panel). Data represent the results of two independent experiments, with 3-4 mice per experiment.

In line with the analysis from the dermis, TIM-4 deficiency did not alter the frequencies of mLCs and CD207^+^ mDDCs in the skin-draining LNs (Figure 4a). The ratio of LN tissue-resident CD207^+^CD8^+^ DCs remained unchanged after the deletion of TIM-4 (Figure 4a). However, lack of TIM-4 nearly doubled the ratios of LN CD207^−^CD4^+^CD8^−^ (25.8 ± 7.1% in WT mice vs. 41.44 ± 4.22% in KO mice, P = 0.029) and CD207^−^CD4^+^CD8^+^ DCs (6.00 ± 0.16% in WT mice Vs 11.04 ± 1.16% in KO mice, P = 0.004) (Figure 4b). Correspondingly, the frequency of CD207^−^CD4^−^CD8^−^ DCs (63.25 ± 8.15% in WT mice vs. 43.06 ± 3.11% in KO mice, P = 0.01) was downregulated in TIM-4 KO mice. Besides, the ratio of CD207^−^CD4^−^CD8^+^ DCs was comparable between TIM-4 WT and KO mice (Figure 4b).

### TIM-4 is not required for LC maturation, phagocytosis and migration

**Figure 5:**
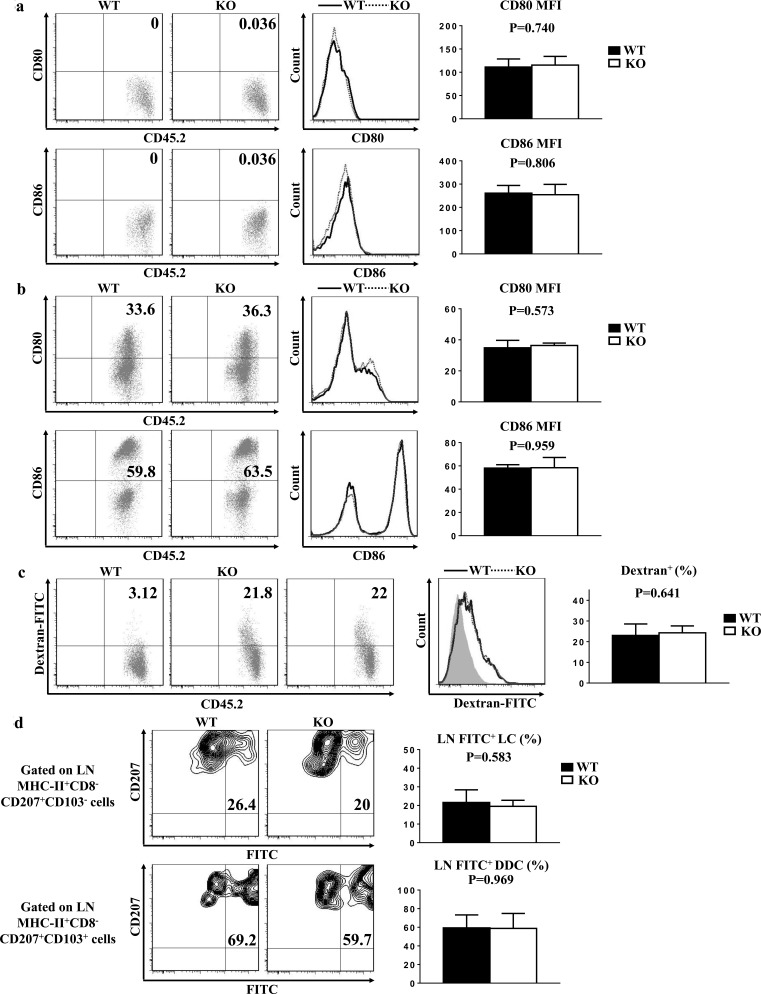
TIM-4 is not required for LC maturation, phagocytosis and migration Epidermal cells were freshly-isolated from the trunk skin of TIM-4 WT and KO mice in (a-c). **a.** Epidermal suspensions were stained with anti-MHC-II, CD45.2, CD80, and CD86 Abs and analyzed by flow cytometry. Data represent two independent experiments, with 4-5 mice per experiment. **b.** Epidermal suspensions from the trunk skin of TIM-4 KO and WT littermates were cultured in complete culture medium for 60 h, and then stained as described in (a). Data represent three independent experiments, with 2-3 mice per experiment. **c.** Epidermal cells were incubated with 0.025% Dextran-FITC for 45 minutes at 37°C or 4°C, which were then stained with anti-CD45.2 and anti-MHC-II. Data represent three independent experiments, with 3-4 mice per experiment (Control: grey filled graph). **d.** TIM-4 WT and KO mice were painted with 200 μl of 5mg per ml FITC in acetone/dibutylphthalate (1:1), and 24 h later the draining LN cells were then stained with anti-MHC-II, anti-CD8, anti-CD207 and anti-CD103. Seven mice were analyzed.

Steady-state epidermal LCs are normally immature, and hardly express co-stimulatory molecules indispensable for LC eliciting T cell priming. Upon maturation, LCs enhance the expressions of these co-stimulatory molecules, including CD80 and CD86. The baseline expressions of CD80 and CD86 in the freshly-isolated epidermal LCs from TIM-4 WT and KO mice were uniformly low (Figure 5a). After 60 hours of *in vitro* culture, the expressions of CD80 and CD86 increased significantly as expected, but the expression levels of CD80 and CD86 in the cultured LCs were comparable between TIM-4 WT and KO mice (Figure 5b). These results indicate that TIM-4 deficiency does not affect the immature status of epidermal LCs and their ability to develop a mature phenotype *in vitro*.

Locating at the outermost skin layer, LCs efficiently capture and process environmental or autologous antigens, which is essential for the induction of immune defense or tolerance [44–46]. To assess the role of TIM-4 in the phagocytic capacity of LCs, freshly-isolated epidermal cells from WT and KO mice were incubated at 37°C or 4°C (as control) with Dextran-FITC for 45 minutes. The ratios of FITC-positive LCs were considerably parallel between WT and KO mice (Figure 5c). Hence, loss of TIM-4 does not impair the antigen-uptaking function of epidermal LCs.

To further evaluate the role of TIM-4 in the phagocytosis and migration capability of epidermal LCs *in vivo*, FITC was applied onto the skin of TIM-4 WT and KO mice as an indicator of antigen capture. There was no significant changes in not only the ratio of MHC-II^high^ mLCs but also the frequency of FITC-positive mLCs (21.6 ± 4.8% in WT mice vs. 19.54 ± 2.91% in KO mice, P = 0.58) (Figure 5d). Similar results were also found in the LN CD207^+^ DDCs (Figure 5d). Consequently, TIM-4 is not required for the *in vivo* phagocytic and migrating abilities of epidermal LCs and CD207^+^ DDCs.

### TIM-4 deficiency does not affect CHS response

**Figure 6:**
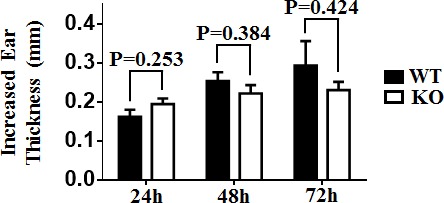
TIM-4 deficiency does not affect contact hypersensitivity response Mice were sensitized with DNFB (0.5%) in the abdominal area and then challenged with DNFB (0.2%) 5 days later to induce delayed type hypersensitivity in the ears. Ear thicknesses were measured at different time points after challenge (24h, 48h, 72h) by comparing challenged and unchallenged ears using a thickness gauge in a blinded manner. Ten mice were analyzed.

Cutaneous DCs have a profound regulatory role in the sensitization phase of CHS response [47]. To determine the potential role of TIM-4 in CHS, we sensitized mice by topically applying 0.5% DNFB on shaved abdominal skin, and then challenged with 0.2% DNFB on day 5. The increased ear thickness induced by the challenge at different time points (24h, 48h, 72h) exhibited no significant differences between TIM-4 WT and KO mice (Figure 6), implying that loss of TIM-4 does not affect CHS response *in vivo*.

### TIM-4 deficiency does not alter LC repopulation

**Figure 7:**
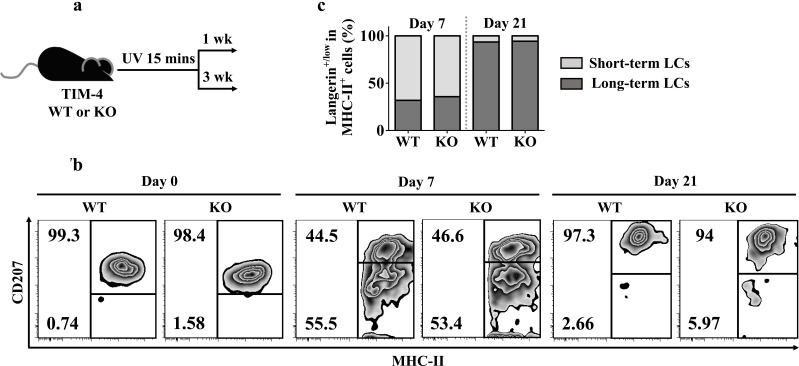
TIM-4 deficiency does not alter LC repopulation **a.** Schematic representation of the experimental plan. **b.**-**c.** The mice were given 15 minutes of UV exposure: **b.** Analysis of the proportions of short-term LCs (MHC-II^+^ langerin^low^) and long-term LCs (MHC-II^+^ langerin^+^) on day 7 and 21 after UV treatment. **c.** Charts represent the frequencies of short-term LCs (MHC-II^+^ langerin^low^) and long-term (MHC-II^+^ langerin^+^) cells from each genotype at different time points. The data represent the results of two independent experiments, with 5-7 mice per experiment.

Recent researches uncovered a unique LC replenishment pattern in inflamed state: short-term LCs (MHC-II^+^ langerin^low^), which derive from circulating Gr-1^hi^ monocytes, and long-term LCs (MHC-II^+^ langerin^+^) of bone marrow (BM) origin would transiently or stably reconstitute the LC compartment, respectively [48]. To explore a possible role of TIM-4 during LC repopulation, we treated TIM-4 WT and KO mice with ultraviolet (UV) for 15 minutes (Figure 7a). As expected, UV exposure led to a transient loss of long-term LCs along with a recruitment of short-term LCs (Figure 7b). Despite the discrepancy in epidermal LC density under steady state, the proportions of short-term LCs (MHC-II^+^ langerin^low^) and long-term LCs (MHC-II^+^ langerin^+^) were equivalent between TIM-4 WT and KO mice both on day 7 and day 21 after UV treatment (Figure 7c). Therefore, TIM-4 does not affect LC replenishment in inflamed state.

## DISCUSSION

Although a potent immunoregulatory role of TIM-4 is gaining attention, the knowledge of TIM-4 on skin-related DCs remains quite limited. To the best of our knowledge, only one study briefly mentioned that in the skin-draining LNs, the TIM-4 expression on migrated CD11c^+^ DCs was upregulated after contact sensitization or phagocytosis of bacteria, suggesting that environmental stimuli could modulate TIM-4 expression on cutaneous DCs [14]. Likewise, enhanced TIM-4 expression together with a mature phenotype has been observed in other types of DCs treated with bacterial toxins. Normally, only a minority of mouse intestinal mucosal DCs expressed TIM-4; however, exposure of staphylococcus enterotoxin B (SEB) increased the expressions of TIM-4 and other co-stimulatory molecules in a dose-dependent manner [15]. Similar results were also found in SEB-treated human circulating DCs and BM-derived DCs (BMDC) stimulated with cholera toxin [49, 50]. Likewise, we have discovered that TIM-4 exhibits a dynamic change from a rather low level in immature epidermal LCs to a moderate level in mature LCs within the skin-draining LNs. Hence, it is possible that invading microbes and relevant products at the skin barrier trigger an upregulation of TIM-4 expression in LCs.

Even though the maturation of LCs was accompanied by an increase of TIM-4 expression, our results demonstrated a redundant role of TIM-4 during LC maturation. Previous research into other DC populations found similar results: blockade of TIM-4 binding by antibodies neither alter the co-stimulatory molecule expressions in mouse splenic DCs [16] nor interfere with the maturation process of human circulating DCs [49]. Intriguingly, although microbial products were potent stimuli of DC maturation, not all of them could simultaneously induce TIM-4 expression [49]. This result implied that TIM-4 was merely involved in selective mechanism of DC activation. In brief, TIM-4 does not affect the maturation capability of DCs, particularly epidermal LCs.

Immature DCs are capable of phagocytosing endogenous antigens such as apoptotic cells [51]. Kushwah *et al* reported that the specific uptake of apoptotic DCs converted viable immature DCs into tolerogenic DCs, which further secreted transforming growth factor-β1 (TGF-β1) and induced Treg differentiation [52, 53]. This result suggested that phagocytosis of apoptotic DCs might be an important cue for viable DCs to elicit immune tolerance. As far as we knew, milk fat globule EGF factor VIII (MFG-E8) had been the only PS receptor identified on epidermal LCs before our study [54]. In contrast to TIM-4, MFG-E8 was highly expressed by immature LCs, and its expression was downregulated upon maturation [54]. Using MFG-E8 null mice, Yoshino *et al* acclaimed that LCs might not principally capture self-antigens in the form of apoptotic cells, as lack of MFG-E8 failed to prevent the accumulation of melanin granules, an indicator of apoptotic keratinocytes [55]. Our data has revealed little contribution of TIM-4 to the general phagocytic capacity of epidermal LCs. We believed that TIM-4 might not be responsible for the engulfment of apoptotic cells by epidermal LCs, given that it was predominantly expressed by mature LCs of compromised phagocytic ability. It is still unclear if epidermal LCs or other cutaneous DCs capture apoptotic cells, especially apoptotic DCs. A comprehensive analysis of other PS receptors among these DC subpopulations might further benefit the understanding of their immunoregulatory roles.

Unlike other BM-derived conventional DCs, adult mouse LCs largely stem from embryonic fetal liver monocytes with a minor contribution of yolk sac (YS)-derived macrophages [56]. Recently, Syrjänen *et al* discovered that the myeloid-potential progenitor cells in the fetal liver expressed a low level of TIM-4, indicating that TIM-4 deficiency might disturb LC homeostasis through direct control of their progenitor cells [57]. However, we cannot exclude the possibility that loss of TIM-4 alters local microenvironment for LC maintenance. Likewise, TIM-4 also regulates the development and homeostasis of LN CD207^−^CD4^+^ DCs. The mechanisms underlying the ratio changes of epidermal LCs and LN CD207^−^CD4^+^ DCs caused by TIM-4 deficiency require future study.

Previous data suggest that TIM-4 predominantly mediates immune repression in the steady state, while it favors a Th2 polarization in response to environmental stimulus [15, 21, 49, 50]. For the first time, we dissected mouse skin-related DC subsets and demonstrated that TIM-4 was preferentially expressed in LCs, CD207^+^ DDCs, LN CD207^+^CD8^+^ and CD207^−^CD4^+^ DCs with distinct patterns, which indicated potential immunoregulatory effects of TIM-4 among these DC subpopulations. As blockade of TIM-4 in splenic DCs enhanced iTreg generation by suppressing the canonical IL-4/ signal transducer and activator of transcription 6 (STAT6)/Gata3 pathway of Th2 lineage, TIM-4 might also regulate LC-mediated skin T cell polarization, especially iTreg and Th2 [16, 58–60]. CD207^+^ DDCs are capable of priming both Th1 and T cytotoxic 1 (Tc1) cells, whereas they could also cross-present antigens to induce tolerance via removal of antigen-specific CD8^+^ T cells [61–64]. The highly-expressed TIM-4 on CD207^+^ DDCs might participate in these immune processes. LN CD207^−^CD4^+^ and CD207^−^CD8^+^ DCs respectively promote Th2 and Th1 cell response, therefore TIM-4 is probably involved in the Th2 polarization elicited by LN CD207^−^CD4^+^ DCs [32]. Intriguingly, early data revealed that splenic CD4^+^ and CD8^+^ DCs displayed a comparable level of TIM-4 expression, in contrast to the preferential TIM-4 expression on CD4^+^ over CD8^+^ conventional DCs within skin draining LNs [14, 18]. This discrepancy suggested that TIM-4 might possess other immune functions in skin-related DCs, for the reason that a majority of published data about TIM-4 utilized splenic DCs.

As a model for allergic contact dermatitis, CHS responses to percutaneous haptens critically depend on T cell priming mediated by skin-related DCs. Most studies demonstrated that LCs induced CHS efficiently, especially at low hapten doses [65–67]. However, constitutive lack of LCs would enhance CHS, implicating a bidirectional role of LCs during immune regulation [68]. Additionally, both CD207^+^ and CD207^−^ DDCs were capable of engulfing haptens at higher concentrations and triggering CHS responses in the absence of LCs [66, 69–71]. In brief, cutaneous DCs display functional redundancy in the elicitation of CHS. Mizui *et al* have exploited CHS model to assess the role of TIM-4 during T-cell mediated immune responses [14]. Interestingly, anti-TIM-4 antibodies, which blocked its binding, enhanced CHS responses when administered during sensitization, whereas diminished ear swelling at the elicitation phase [14]. Paradoxically, we demonstrated here that loss of TIM-4 did not affect CHS responses. The underlying causes for these divergent findings may include: (i) the former study utilized blocking antibodies against TIM-4, that might possess unknown off-target effects, while we used TIM-4 KO mice in which TIM-4 gene was specifically deleted; (ii) the distinct subpopulations of skin-related DCs expressed TIM-4 at different levels, which may not be completely as well as uniformly blocked by anti-TIM-4 antibodies; (iii) TIM-4 deficiency throughout lifetime altered the properties of multiple immunocytes, including T cell hyperaction, which may disguise the effects mediated by skin-related DCs.

In conclusion, for the first time we have revealed the *in vivo* expression patterns of TIM-4 in the distinct subsets of DCs in skin and skin-draining LNs, and discovered that TIM-4 deficiency upregulated the frequencies of epidermal LCs and LN CD207^−^CD4^+^ DCs. Future research addressing the specialized function of TIM-4 in each skin-related DC subpopulations might provide novel strategies to modulate immune responses under various conditions, which include infection, allergy, neoplasm, autoimmunity and vaccination.

## MATERIALS AND METHODS

### Mice

TIM-4^−/−^ C57BL/6 knock-out (TIM-4 KO) mice have been described previously [21], kindly provided by Dr. Vijay K. Kuchroo (Brigham and Women's Hospital, Harvard Medical School). C57BL/6 wild-type (TIM-4 WT) mice were purchased from the Jackson Laboratory. Experiments were conducted at 6 to 12 weeks of age. Mice were housed in a specific pathogen-free barrier unit. Handling of mice and experimental procedures were in accordance with the requirements of Institutional Animal Care and Use Committee.

### Skin single-cell suspension preparations

Epidermal and dermal cell suspensions were prepared from the trunk skin of TIM-4 WT and KO mice, as previously reported [72, 73]. In brief, trunk skin was incubated in 0.5% dispase (Gibco, Life Technologies, Grand Island, NY, USA) for 1 hour at 37°C after its subcutaneous fat was scraped off. Then, epidermal sheet was peeled from the dermis, cut into tiny pieces and digested in complete culture medium containing 0.01% DNase (Sigma, St Louis, MO, USA) for 1 hour at 37°C. The underlying dermis was cut into approximately 3 × 3 mm pieces, which were floated in 0.04% Liberase TL (Roche Diagnostics GmbH, Mannheim, Germany) for 45 minutes and subsequently 0.04% Liberase TL + 0.005% DNase for additional 15 minutes at 37°C. The epidermal and dermal single-cell suspensions were harvested after filtering through a 70 μM filter. Complete culture medium was RPMI 1640 (with 2mM L-glutamine, Gibco) supplemented with 10% heat-inactivated fetal bovine serum (Hyclone, Thermo Scientific, Pittsburgh, PA, USA), 5 × 10 ^5^ M 2-mercaptoethanol, 0.15% sodium hydrogencarbonate, 1 mM sodium pyruvate, nonessential amino acids, 100U per ml penicillin and 0.01% streptomycin.

### Flow cytometry and antibodies

Single-cell suspensions were pretreated with anti-FcγRII/III (clone 2.4G2) for 10 minutes at 4°C, and then stained for surface and intracellular markers with the conjugated monoclonal antibodies listed as below: I-A/E (MHC-II) (M5/114.15.2), CD45.2 (104), TIM-4 (RMT4-54), CD4 (RM4-5), CD8 (53-6.7), CD103 (2E7), CD11c (N418), CD11b (M1/70), CD80 (16-10A1), CD86 (GL1) and CD207 (929F3.01). All antibodies were purchased from eBioscience (San Diego, CA, USA) or Dendritics (Lyon, France). Cells were analyzed with a BD LSR II flow cytometer using FACS Aria II (BD Biosciences, San Jose, CA, USA) and FlowJo software version 7.6.1 for Microsoft (TreeStar, Sam Carlos, CA).

### Phagocytosis assay

Freshly-isolated epidermal cells were incubated with 0.025% Dextran-Fluorescein Isothiocyanate (FITC) (Life Technologies, Grand Island, NY, USA) for 45 minutes at 37°C or 4°C. Then, cells were washed by phosphate buffered saline (PBS), and subsequently stained with anti-CD45.2 and MHC-II, the percentage of LCs that uptake antigen (CD45.2^+^/MHC-II^+^/FITC^+^) was determined by flow cytometry [74, 75].

### *In vitro* maturation

Freshly-separated epidermal cells were cultured with complete culture medium at 37°C for 60 hours. The cells were then collected and stained with anti-MHC-II, CD207, CD45.2, CD80 and CD86 for flow cytometric analysis [76].

### Hapten sensitization and elicitation of CHS

On day 0, mice were sensitized by applying 25μl of 0.5% DNFB (Sigma-Aldrich, St. Louis, MO, USA) (acetone:olive oil = 4:1) on shaved belly skin. On day 5, sensitized mice were challenged topically with 10μl of 0.2% DNFB on the left ear, whereas 10μl of acetone/olive oil (4:1) was painted on the right ear. Ear thicknesses were measured by comparing challenged (left) and unchallenged (right) ears using a thickness gauge (Digimatic caliper, Mitutoyo, Japan) in a blinded manner. And, ear thickness increases were calculated by subtracting pre-challenge (0h) from post-challenge measurements (24h, 48h, 72h).

### LC replenishment under skin inflammation

For the depletion of LCs, mice were exposed to ultraviolet (UV) for 15 minutes (wavelength 254 nm, voltage 8W, source: 38 cm). The replenishment of the LC network was assessed 1 week and 3 weeks after UV treatment.

### Statistical analysis

Data were presented as mean ± standard deviation (SD). Statistics analyses were performed with GraphPad Prism software (GraphPad, San Diego, CA, USA) using a two-tailed Student t test. Differences were considered to be statistically significant when P < 0.05.
